# Impact of the Bolsa Família program on food availability of low-income Brazilian families: a quasi experimental study

**DOI:** 10.1186/s12889-016-3486-y

**Published:** 2016-08-19

**Authors:** Ana Paula Bortoletto Martins, Carlos Augusto Monteiro

**Affiliations:** 1 Programa de Pós-graduação em Nutrição em Saúde Pública, Faculdade de Saúde Pública, Universidade de São Paulo, Av. Dr. Arnaldo, 715 São Paulo, SP Brazil; 2 Núcleo de Pesquisas Epidemiológicas em Nutrição e Saúde, Faculdade de Saúde Pública, Universidade de São Paulo, São Paulo, Brazil; 3 Departamento de Nutrição, Faculdade de Saúde Pública, Universidade de São Paulo, São Paulo, Brazil

**Keywords:** Income transfer, Food availability, Propensity score

## Abstract

**Background:**

The *Bolsa Família* Program was created in Brazil in 2003, by the joint of different social programs aimed at poor or very poor families with focus on income transfer to promote immediate poverty relief, conditionalities and complementary programs. Given the contributions of conditional cash transfer programs to poverty alleviation and their potential effects on nutrition and health, the objective of this study was to assess the impact of the *Bolsa Família* Program on food purchases of low-income households in Brazil.

**Methods:**

Representative data from the Household Budget Survey conducted in 2008–2009 were studied, with probabilistic sample of 55,970 households. 11,282 households were eligible for this study and 48.5 % were beneficiaries of the BFP. Food availability indicators were compared among paired blocks of households (*n* = 100), beneficiaries or non-beneficiaries of the *Bolsa Família* Program, with monthly *per capita* income up to R$ 210.00. Blocks of households were created based on the propensity score of each household to have beneficiaries and were homogeneous regarding potential confounding variables. The food availability indicators were weekly *per capita* expenditure and daily energy consumption, both calculated considering all food items and four food groups based on the extent and purpose of the industrial food processing. The comparisons between the beneficiaries and non-beneficiaries blocks of households were conducted through paired ‘t’ tests.

**Results:**

Compared to non-beneficiaries, the beneficiaries households had 6 % higher food expenditure (*p* = 0.015) and 9.4 % higher total energy availability (*p* = 0.010). It was found a 7.3 % higher expenditure on *in natura* or minimally processed foods and 10.4 % higher expenditure on culinary ingredients among the *Bolsa Família* Program families. No statistically significant differences were found regarding the expenditure and the availability of processed and ultra-processed food and drink products. In the *in natura* or minimally processed foods group, the expenditure and the availability of meat, tubers and vegetables were higher among the *Bolsa Família* Program beneficiaries.

**Conclusion:**

The *Bolsa Família* Program impact on food availability among low-income families was higher food expenditure, higher availability of fresh foods and culinary ingredients, including those foods that increase diet’s quality and diversity.

## Background

Conditioned cash transfer programs (CCTP) were conceived with the purpose of expanding the guarantee of social protection, fighting poverty and reducing social inequalities. They are based on monthly cash transfers to low-income families, conditioned to the beneficiaries’ fulfillment of an agenda in the areas of health, education and social services [1, 2].

In Brazil, the conditioned cash transfer programs were created in the 1990’s. However, their expansion only occurred from 2001, with the development of the federal programs “Bolsa Escola”, “Bolsa Alimentação”, “Auxílio Gás” and “Cartão Alimentação”. Since 2003, these programs’ resources were gradually centralized in one program, the *Bolsa Família* Program (BFP). The BFP has three main focus: the income transfer to promote immediate poverty relief; conditionalities that reinforce the access to basic social rights in education, health and social care; and complementary programs aimed at the families development, so that they are able to overcome the vulnerability.^1^

By the end of 2009, families eligible to participate in the BFP were those with a monthly *per capita* income lower than R$70.0 (US$28.0), regardless of the household’s composition, or with a monthly income lower than R$140.00 (US$56.0) with children, teenagers, pregnant women or nursing mothers. Benefits ranged from R$68.00 (US$27.2) to R$200.00 (US$80.0) per family. In 2012, the *Bolsa Família* Program benefited 13.9 million households, corresponding to the total eligible families in poverty situation estimated by the 2010 Brazilian Demographic Census.^2^

The contribution of CCTP has been evaluated as positive, especially regarding the reduction of social inequalities. In Brazil, there was a decrease of 4.6 % in the Gini coefficient between 2001 and 2005, which was higher than in other countries’ [3, 4]. Positive impacts were also observed regarding health outcomes such as infant mortality and immunization [5, 6].

Impact evaluation studies of the CCTP on diet and nutrition of the beneficiary families in Brazil were systematically reviewed and indicate positive results, but they were considered fragile regarding to the sampling strategy (frequently, not representative samples of the population), the study design and the data analysis (without adjustments for confounding variables). These findings prevent to make a robust evaluation of the programs impact [7].

Regarding food consumption, the food industrial processing, a component that has impacts upon population’s health, has been practically ignored in the CCTP evaluations. In 2010, a classification based on the extension and purpose of food processing was published, in order to provide a deeper understanding of the modifications in food systems. The relevance and implications of food processing in tackling obesity and other chronic diseases have been reported [8–12].

Given this scenario, it was found necessary to conduct an impact evaluation of the BFP on the diet of the Brazilian population, with rigorous methodological approach, in order to provide high-quality evidence for public policies decision making. The aim of this study was to evaluate the impact of the conditioned cash transfer program *Bolsa Família* on food purchases of low-income families in Brazil.

## Methods

The database of the Household Budget Survey (HBS) conducted by the Brazilian Institute of Geography and Statistics^3^ in 2008–2009 was employed for this study. This research used a complex cluster sampling in two stages. It included the geographical and socioeconomic stratification of the country’s census tracts, followed by random drawings of sectors in the first stage and of households in the second stage.^4^

Low-income households, with monthly *per capita* income of less than R$210.00 were selected for the study. This value is equivalent to the cutoff point for inclusion of families in the BFP (R$140.00 *per capita*) plus 50 %, to compensate for imprecisions in the income information. This decision was made to avoid the exclusion of people receiving BFP with incomes higher than the cutoff, and the value of 50 % was determined after consistency analysis comparing the household income and the declared income from social programs. From the individual income records of the HBS, the “beneficiaries of the *Bolsa Família* Program” were identified as all households in which a resident declared to receive any monetary value from the *Bolsa Família* Program for the period of 12 months prior to data collection. All other households were considered non beneficiaries. Of the total of 55,970 households studied in 2008–09, 11,282 households were eligible for this study and 48.5 % were beneficiaries of the BFP.

The study design was quasi-experimental, since the households were not initially randomized in groups of BFP beneficiaries and non-beneficiaries. Nevertheless, we paired groups of households according to propensity scores, a procedure that matches the two groups, considering characteristics that could influence household food availability [13, 14].

The main information analysed were the purchases of food items for household consumption by the families for seven consecutive days, recorded daily in a notebook, by household members or IBGE interviewer if necessary. Data collection was distributed among the census tracts over 12 months uniformly among the survey strata, ensuring representation in the four quarters of the year.

The food purchased for consumption outside the home were not recorded with sufficient detail and were excluded from analysis. The HBS collected information of monetary and non-monetary food acquisition, such as donation, self-production or exchange. In the last case, an estimated value of the acquisition (in Reais) was imputed in the database.

Total quantities of each food item, after excluding the inedible portions,^5^ were converted into energy, using the Brazilian Table of Food Composition^6^ and the USDA Food Composition Table.^7^ Total daily *per capita* energy was calculated by the sum of each food item calories divided by the number of dwellers and the seven days of the survey.

All food items were grouped according to the NOVA classification based on the extent and purpose of industrial food processing [15, 16]. The items were grouped into four groups and subgroups: *In natura* or minimally processed foods (Group 1), which are natural foods altered by processes such as removal of inedible or unwanted parts and no addiction of substances such as salt, sugar and/or oils or fats. Group 1 includes rice, beans, cassava flour, wheat flour, pasta, meat, milk, eggs, fish, fruits, vegetables, roots and tubers and other foods; Processed culinary ingredients (Group 2) consisted of substances obtained directly from group 1 foods or from nature by processes such as pressing, refining milling etc. and are usually used in home or restaurant kitchens to prepare, season and cook group 1 foods. Group 2 includes sugar, salt, vegetable oil, animal fat; Processed food products (Group 3) are foods with added substances such as salt, sugar, or oil. Group 3 includes canned or bottled vegetables, fruits and legumes, salted, cured or smoked meat, canned fish, fruits in syrup; Ultra-processed food and drink products (Group 4) which are industrial formulations typically with five or more ingredients and substances not commonly used in culinary preparations and typically contain little or no Group 1, developed with the purpose to create ready to eat, to drink or to heat products, liable to replace the consumption of Group 1 and Group 2 foods. Ultra-processed food and drink products are carbonated drinks, sweet or savory snacks, ice-creams, chocolates, margarines and spreads, cookies, breakfast cereals, cocoa drinks, ready to heat products such as pizza or pasta dishes, poultry and fish ‘nuggets’ and ‘sticks’, ‘instant’ soups, noodles etc. The detailed description of NOVA classification can be found elsewhere [16].

To describe the beneficiaries and non-beneficiaries households, the mean values of the following socio-demographic and economic characteristics were calculated: monthly *per capita* income, proportion of food expenditure outside the household, schooling of the head of the family, number of people in the family, *per capita* number of rooms, *per capita* number of bathrooms, proportion of households with piped water and the distribution of individuals by gender and age groups. It was calculated the proportion of households in each of the five Brazilian regions (North, Northeast, Southeast, South and Middle-East) and in the urban (separated for State capitals and other cities) and rural areas.

For the beneficiary households, it was calculated the benefit’s monthly *per capita* value and its share in the total *per capita* income (%). The participation period was informed by the beneficiaries.

To conduct the descriptive analysis, the statistical significance of the differences was obtained by chi-squared test (*χ*^2^) with Yates correction (for the difference of proportions) and by test for difference of means for independent samples (Student *T*-test).

The propensity score matching method was applied [13] to reduce the possible biases in estimating the BFP impacts on food acquisition with data from an observational study. The Stata application ‘pscore.ado’ [17] was used to estimate the probability of each household to belong to the BFP, according to the steps described below.

The first step was to calculate the propensity score of each household be a beneficiary of the BFP. We conducted a probit regression model, with the variable “beneficiary or not of the program” as the outcome. The characteristics included were: monthly *per capita* income, schooling of the household head, number of rooms *per capita,* number of bathrooms *per capita,* presence of piped water, number of people per household, share of household spending on food outside the house, region, area and distribution of residents by sex and age (0–9, 10–15, 16–20, 21–65 and above 65 years old).

**Fig. 1:**
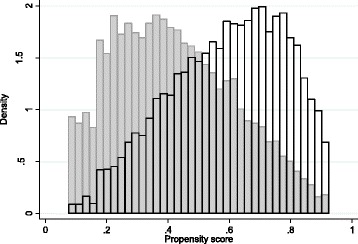
Distribution of the households’ density, according to the propensity score and belonging to the *Bolsa Família* Program (in gray: non-beneficiaries; blank: beneficiaries)

The average propensity score range was from 0.008 to 0.958. The distribution of beneficiary or non-beneficiary households is presented in Fig. 1. There was an overlap between households with similar propensity score, a necessary condition to obtain a proper matching.^8^

The second step was to perform the pairing of households with similar propensity score by the nearest neighbour procedure. Due to the short period of reference for data collection on food items acquisition (only seven days of the month), it was not possible to conduct the analysis on the household level, because of the monthly food acquisition variation according to the week. To overcome this survey characteristic, households were grouped into blocks of beneficiaries and non-beneficiaries of the BFP, according to the value of the propensity score of each household. The mean propensity score of each pair of beneficiaries or non-beneficiaries households blocks were compared statistically. If a significant difference was obtained, the block was divided and the test redone until obtaining pairs of households blocks beneficiaries and non-beneficiaries of the BFP with no significant differences in the mean propensity score [17]. As a result, we identified 117 pairs of blocks of households beneficiaries and non-beneficiaries of the BFP and with similar propensity scores.

Finally, we tested the balance of the pairs of household blocks. The balancing consisted in statistically compare the blocks for each of the variables of the probit model (socio-economic and demographic characteristics and household conditions), in order to obtain pairs of homogeneous blocks regarding potential confounders in the relationship between belonging to the BFP and food acquisition. Non-balanced blocks or those with less than four households were excluded from the analysis. Among the non-beneficiaries blocks, the mean number of households was 53.9, and among the beneficiaries’ blocks, the mean value was 55.1. After pairing and balancing the blocks, 2.8 % of households were excluded. It was not found any statistically differences between socioeconomic and demographic characteristics of the excluded and selected households (Data not shown).

In order to establish the BFP impact on food purchases, we compared the mean *per capita* values between the beneficiaries and non-beneficiaries household blocks of two indicators: the weekly spending (R$) and the daily energy availability (kcal), both relative to all food items and to each of the food groups and subgroups studied. To obtain the total weekly *per capita* expenses with food, the value of each expenditure on every food item was added and divided by the number of dwellers in each household. Statistical significance of the comparisons between pairs of beneficiaries and non-beneficiaries household blocks was assessed with paired ‘t’ of Student tests.

The statistic package Stata SE v.12.1 was employed for all analyses. The significance level of 5 % was adopted for all statistical tests.

This study was approved by the Research Ethics Committee, Faculty of Public Health of the University of São Paulo.

## Results

Beneficiaries households of the BFP received, on average, R$20.20 *per capita* per month, ranging from R$0.30 to R$141.70. The benefit share on monthly income varied from 0.2 to 100.0 %. The mean period of participation in the program was 11.3 months (SE: 0.0 months). Beneficiary households had lower monthly *per capita* income and lower educational level, lower proportion of bathrooms, rooms *per capita* and piped water than non-beneficiary households, as well as lower food spending outside the house. The age and sex distribution indicates a higher proportion of young people of both genders in the beneficiary households. Regarding the geographical distribution, beneficiary households were located more commonly in the Northeastern region and in rural areas of Brazil. The opposite situation was found for the location in the Southeast, South and Center-West regions and in urban areas of the country. In the Northern region, the proportion of households was similar between the two groups (Table 1).

**Table 1:** Characterization of *Bolsa Família* Program beneficiaries and non-beneficiaries’ households, according to socioeconomic and demographic variables, region and area of residence. Brazil, 2008–09

	Non-beneficiaries (*n* = 5,455)		Beneficiaries (*n* = 5,517)		
Characteristics	Mean/Frequency	SE	Mean/Frequency	SE	p*
*Per capita* monthly income ^a^(R$)	140.1	1.0	116.3	1.0	<0.001
Years of education of head of household	4.7	0.1	3.5	0.1	<0.001
Number of persons per household	4.4	0.0	5.0	0.0	<0.001
Number of bathrooms *per capita*	0.3	0.0	0.2	0.0	<0.001
Number of rooms *per capita*	1.3	0.0	1.1	0.0	<0.001
Presence of running water (%)	80.0	0.7	70.8	0.8	<0.001
Spent on food away from home (%)	15.3	0.6	13.1	0.4	<0.001
Distribution of subjects by sex and age (%)					
Males from 0 to 9 years	13.0	0.3	13.4	0.3	0.013
Females from 0 to 9 years	11.8	0.3	12.9	0.3	<0.001
Males from 10 to 15 years	6.8	0.3	10.2	0.3	<0.001
Females from 10 to 15 years	6.2	0.2	9.3	0.2	<0.001
Males from 16 to 20 years	4.7	0.2	4.8	0.2	0.368
Females from 16 to 20 years	5.3	0.2	4.1	0.2	<0.001
Males from 21 to 65 years	24.0	0.3	19.7	0.2	<0.001
Female from 21 to 65 years	26.5	0.3	24.7	0.2	<0.001
Males over 65 years	0.9	0.1	0.4	0.0	<0.001
Females over 65 years	1.0	0.1	0.5	0.1	<0.001
Region (%)					
North	12.3	0.5	11.3	0.4	<0.001
Northeast	43.6	0.9	65.7	0.9	
Southeast	26.6	1.1	15.0	0.9	
South	9.7	0.6	4.5	0.4	
Midwest	7.7	0.4	3.5	0.2	
Area (%)					
Urban (capital)	16.4	0.9	9.6	0.6	<0.001
Urban (other cities)	55.2	1.0	50.3	0.9	
Rural	28.4	0.8	40.1	0.9	

Excluded households with *per capita *income above R$ 210.00

*SE* standard error

*p values for Student *T*-test for all variables except for region and area, in which the chi-squared test (*χ* ^2^) with Yates correction was applied

^a^values after excluding the benefit in the case of families included in the program

The beneficiary and non-beneficiary household blocks were similar in almost all the variables used to construct the propensity scores, except for the proportion of food spending outside the household, which was higher among the BFP beneficiaries (data not shown).

Regarding the impact of BFP on food purchases, the total weekly *per capita* expenditure on food was significantly higher, at around 6 % (R$0.63), among the beneficiaries’ household blocks. The expenditure on *in natura * or minimally processed foods (Group 1) of the beneficiaries was 7.7 % higher than the non-beneficiaries, and on culinary ingredients (Group 2), the expenditure was 18 % higher. There were no differences in the processed (Group 3) and ultra-processed products (Group 4) expenses (Table 2). Among Group 1, a greater spending on fresh meats, roots, tubers and vegetables and other *in natura* or minimally processed foods and in Group 2, a higher spending on sugar, vegetable oil and other culinary ingredients were found between beneficiaries’ household blocks. Regarding the ultra-processed products, the non-beneficiary household blocks had higher spending on confectionary and other sugar based products (Table 3).

**Table 2:** Weekly *per capita* expenditure (in R$^c^) of the *Bolsa Família* Program beneficiaries and non-beneficiaries households blocks, according to the food groups. Brazil, 2008–09

	Non-beneficiaries (*n* = 100)		Beneficiaries (*n* = 100)		
Food items	Mean	SE	Mean	SE	*p* ^*^
*In natura* or minimally processed foods	**6.75**	**1.31**	**7.27**	**1.57**	**0.004**
Rice	0.85	0.30	0.94	0.43	0.082
Beans	0.49	0.16	0.52	0.25	0.302
Pasta	0.19	0.07	0.19	0.09	0.894
Wheat flour	0.06	0.05	0.07	0.09	0.280
Manioc flour	0.26	0.20	0.27	0.15	0.627
Fruits	0.39	0.19	0.40	0.19	0.583
Vegetables	0.33	0.14	0.37	0.17	**0.022**
Roots and tubers	0.10	0.06	0.13	0.10	**0.001**
Milk	0.63	0.23	0.62	0.30	0.783
Meat	2.40	0.66	2.59	0.72	**0.021**
Fish	0.43	0.26	0.45	0.27	0.634
Eggs	0.18	0.07	0.20	0.09	0.209
Other *in natura* or minimally processed foods^a^	0.45	0.17	0.52	0.20	**0.012**
Processed culinary Ingredients	**0.74**	**0.21**	**0.88**	**0.44**	**0.009**
Sugar	0.30	0.11	0.35	0.15	**0.007**
Salt	0.03	0.02	0.03	0.02	0.583
Condiments and seasonings	0.05	0.03	0.05	0.05	0.662
Oils	0.29	0.10	0.36	0.32	**0.037**
Animal fats	0.03	0.02	0.03	0.02	0.367
Other processed culinary ingredients^b^	0.05	0.04	0.06	0.04	**0.009**
Processed Products	**0.92**	**0.22**	**0.95**	**0.33**	**0.504**
Bread	0.58	0.16	0.60	0.23	0.351
Cheese	0.07	0.07	0.06	0.07	0.427
Processed meat	0.20	0.12	0.21	0.18	0.661
Preserved meat	0.03	0.03	0.03	0.04	0.709
Preserved vegetables	0.02	0.05	0.01	0.02	0.064
Other processed products	0.02	0.02	0.02	0.03	0.735
Ultra-processed products	**1.80**	**0.73**	**1.75**	**0.75**	**0.504**
Ultra-processed breads	0.05	0.04	0.05	0.06	0.468
Bakery products	0.23	0.10	0.25	0.10	0.069
Confectionary and other sugar based products	0.13	0.10	0.10	0.08	**0.022**
Salty snacks	0.17	0.07	0.18	0.08	0.524
Sodas	0.22	0.13	0.22	0.14	0.671
Other sugar sweetened beverages	0.14	0.08	0.15	0.11	0.475
Ultra-processed meats	0.28	0.14	0.25	0.14	0.061
Ready-to-eat meal or dishes	0.20	0.16	0.18	0.22	0.655
Sauces and spreads	0.09	0.06	0.09	0.08	0.868
Morning cereals	0.10	0.07	0.10	0.08	0.992
Margarine	0.10	0.05	0.10	0.05	0.722
Total	10.22	2.08	10.85	2.52	**0.015**

*p value for paired Student *T*-test

^a^nuts and seeds, teas and coffee, other legumes, other cereals, soy protein, other flours

^b^other sugars, vinegar, coconut milk, cream

^c^R$ = Brazilian Real -US$1,00 ≈ R$2,50 (in 2009). In Bold, mean values for the four main Groups

**Table 3:** Differences in weekly *per capita * food expenditure (in R$^a^) and *per capita* daily energy availability between the *Bolsa Família* Program beneficiaries and non-beneficiaries’ households blocks, according to food groups. Brazil, 2008–09

	Mean	SE	95 % CI	
Weekly *per capita* expenditure (R$^a^)				
*In natura* or minimally processed food	0.52	0.18	0.17	0.87
Processed culinary ingredients	0.13	0.05	0.03	0.23
Processed Products	0.02	0.04	−0.05	0.10
Ultra-processed products	−0.05	0.07	−0.18	0.09
Total *per capita* expenditure	0.63	0.25	0.12	1.13
*Per capita *daily energy (kcal)				
*In natura* or minimally processed food	53.82	24.32	5.56	102.08
Processed culinary ingredients	51.13	17.07	17.26	85.00
Processed Products	5.46	4.79	−4.05	14.97
Ultra-processed products	5.74	4.46	−3.10	14.59
Total *per capita* energy	115.54	39.40	37.37	193.71

^a^R$ = Brazilian Real - US$1.00 ≈ R$2.50 (in 2009)

Regarding the BFP impact on the mean *per capita* calories, it was found that the beneficiaries had 115.5 kcal higher total food availability: the major part of this purchase was of *in natura* or minimally processed foods (Table 4). The comparisons of the food items quantities (kcal/capita) showed a significantly higher purchase among beneficiaries for total food items (9.7 %), for meat (13.8 %), roots and tubers (40.4 %) and vegetables (15.2 %) in the group of fresh or minimally processed foods. In the group of culinary ingredients, it was found near 20 % higher purchases for vegetable oils and sugar. Among the ultra-processed products, the acquisition of sweet bakery products was significantly higher and the acquisition of ultra-processed meats was 10 % lower in the beneficiary households (Table 4).

**Table 4:** Availability of *per capita* daily energy (in calories) of the *Bolsa Família* Program beneficiaries and non-beneficiaries household blocks, according to the food groups. Brazil, 2008–09

	Non-beneficiaries (*n* = 100)		Beneficiaries (*n* = 100)		
Food items	Mean	SE	Mean	SE	p^*^
*In natura* or minimally processed foods	**692.7**	**174.4**	**746.5**	**171.2**	**0.029**
Rice	243.8	115.2	257.5	117.6	0.398
Beans	74.5	28.3	81.4	41.1	0.158
Pasta	32.0	13.3	31.9	14.3	0.968
Wheat flour	16.8	16.6	20.1	25.9	0.236
Manioc Flour	92.7	88.5	97.3	55.7	0.640
Fruits	20.5	21.1	18.4	10.1	0.355
Vegetables	4.8	2.1	5.5	2.7	**0.024**
Roots and tubers	7.7	6.7	10.9	9.0	**0.006**
Milk	39.0	14.2	40.8	19.5	0.381
Meats	96.5	25.4	109.8	31.4	**0.000**
Fish and other seafood	14.2	9.5	14.4	9.2	0.856
Eggs	7.7	3.0	8.6	4.7	0.099
Other *in natura* or minimally processed foods^a^	42.6	43.5	50.0	40.0	0.202
Processed culinary Ingredients	**286.5**	**88.0**	**337.7**	**142.9**	**0.003**
Sugar	149.9	52.7	175.3	75.4	**0.008**
Condiments and seasonings	1.4	1.1	1.5	1.4	0.516
Oils	122.0	42.7	144.3	84.3	**0.020**
Animal fats	4.8	5.4	4.3	5.2	0.458
Processed Products	**81.5**	**22.1**	**86.9**	**45.6**	0.257
Bread	66.6	19.1	67.5	21.8	0.727
Cheese	3.0	3.1	2.7	3.0	0.503
Processed meat	7.8	6.0	12.3	37.6	0.253
Preserved meat	1.1	1.1	1.1	1.5	0.956
Preserved vegetables	0.8	3.6	0.4	0.9	0.275
Other processed products^b^	1.1	1.3	1.3	2.0	0.407
Ultra-processed products	**129.1**	**38.4**	**134.8**	**39.4**	0.201
Ultra-processed breads	4.2	3.8	4.5	4.4	0.512
Bakery products	23.0	9.2	26.0	9.4	**0.011**
Confectionary and other sugar based products	8.1	6.3	7.2	6.2	0.260
Salty snacks	20.3	8.1	21.8	9.4	0.212
Sodas	8.2	4.5	8.4	5.5	0.671
Other sugar sweetened beverages	2.1	1.6	2.4	2.4	0.308
Ultra-processed meats	21.2	10.5	18.8	8.2	**0.046**
Ready-to-eat meal or dishes	10.4	6.6	11.2	14.4	0.586
Sauces and spreads	2.0	1.7	2.2	3.9	0.546
Morning cereals	11.3	11.2	13.8	12.7	0.143
Margarine	16.8	7.6	17.1	8.6	0.747
Total	1190.0	261.3	1305.5	310.8	**0.004**

*P value for paired Student *T*-test

^a^nuts and seeds, teas and coffee, other legumes, other cereals, soy protein, other flours

^b^other sugars, vinegar, coconut milk, cream In Bold, mean values for the four main Groups

## Discussion

In a study based on a representative sample of the Brazilian population it was observed that the *Bolsa Família* Program has contributed for higher *per capita* expenditure on food, higher *per capita* availability of total calories and higher availability of *in natura* or minimally processed foods and processed culinary ingredients among low-income households.

The increase in food availability is positive for two main reasons. First, the energy availability among low-income families in Brazil is below the national average (1.611 kcal) [18]. Second, the increased availability of food items such as meat, roots, tubers and vegetables can diversify and improve the nutritional quality and palatability of the diet. The consumption of these food items are recommended by the Dietary Guidelines for the Brazilian population as part of a healthy and adequate diet [19].

It is also positive the finding that the participation in the BFP did not influence the purchase of processed foods or ultra-processed food products. The Brazilian low-income population still buys a small amount of these products and the benefit was not primarily used for their purchase. In fact, Brazil is a developing country which still has a food system based on fresh or minimally processed foods and culinary preparations, unlike developed countries like Canada and the UK, where the share of ultra-processed products represents more than 60 % of total calories [9, 20]. On the other hand, the increase in the caloric share of these products in the last decades in Brazil has occurred in all income levels. In fact, the increasing purchasing power of the population was one of the main targets of aggressive marketing strategies from the ultra-processed products industry, as a consequence of the “saturation” of the market in developed countries [21, 22].

The subtle effects of BFP on the dietary quality of the beneficiary families indicate that, by itself, only income increases are not enough to promote substantial improvements in the diet. Besides the income transfer, it is necessary to promote food and nutrition security through public policies that include actions to encourage and guarantee the consumption to healthy foods. One of the problems, for example, is that low income Brazilian families living isolated or distant from the central areas of urban centers have difficulties buying certain foods at affordable prices [23–25].

There are few studies evaluating the impact of CCTP in Brazil. The most similar study was an analysis of BFP impact on food acquisition, with 2008–09 HBS data. It analyzed the annual spending on food of households with income between R$69.00 and R$171.00 and concluded that the beneficiary families had higher annual expenditure on food, mainly of grains, cereals, vegetables, poultry, eggs, bakery, oils and fats.^9^ Another evaluation of the BFP impact on food expenditures in 2005, in rural Northeast, found a significant increase in annual spending on food among beneficiary families [26].

Other studies assessed the association between the BFP with food consumption in Brazil. Two were cross-sectional: one with national representative data of BFP beneficiaries that assessed the perception of change in food consumption after receiving the benefit [27]; and the other assessed the frequency of consumption of three food groups amongst 119 children in the Northeast. [28] A third one was a longitudinal study with 20 women from the Northern rural area. [29] Despite the wide variety of methodologies and lack of representativeness of the last two studies, the results pointed to the increasing diversity of the diet, but also a higher intake of sweet biscuits, candies, chocolates and soft drinks among beneficiary children.

Other two qualitative studies pointed out the importance of the BFP in ensuring the food access, highlighted the vulnerability of this social group and their restricted access to information about nutrition and to more expensive types of foods [30, 31].

In 2005, the Federal Government conducted an impact assessment of the BFP comparing health indicators and household expenditure. It was found a higher household expenditure with foods among the beneficiaries, but no significant differences in spending on ‘basic’ and ‘non basic’ food.^10^

Compared to the aforementioned studies, this was the first study to provide an impact evaluation of the BFP on the amount and the quality of food purchases of the beneficiaries using an appropriate methodological design for the selection of the control group, with adjustment for confounders and with representative data of the population.

The CCTP impact evaluations in Latin America indicate an increase in the amount of food consumed and improvements in diet diversity. In Colombia, there was an increase in energy consumption and of meat, milk, cereals, oils and fats among the beneficiaries of the program “Familias en Acción” [32]. The Mexican program promoted a 6.4 % increase in caloric availability between beneficiary households and also increased consumption of vegetables and animal products [33]. In Nicaragua, the beneficiary families from the program “Red de protección social” declared higher consumption of beans in the poorest regions, and occasional purchases of meat, in the less poor regions [34].

Some of the study limitations are related to the data source. The effective individual food consumption was not assessed, therefore it is not possible to estimate the fraction of food purchased and not consumed, neither if the products were shared with others than the family members. However, there is no reason for substantial differences in food waste or food sharing in beneficiary and non-beneficiary households. Also, the HBS does not assess with sufficient detail the consumption of food outside the house, which represented 18 % of the energy consumed in 2008–09 [35]. Nevertheless, this spending among low-income families equals about half the national average and this information was included as a confounding variable. Finally, there is the short period for recording the household’s food and drink acquisition, which was contoured by employing blocks of households as study units.

Another limitation is the lack of randomization in the selection of households included or not in the BFP. The propensity score matching identified beneficiaries and non-beneficiaries households, with similar propensity to belong to the BFP, taking into account a large number of variables that potentially could influence food purchases. However, there is no guarantee that all relevant variables were considered. It’s worth noting that the definition of belonging to BFP is consistent with the information of the average time of receiving the benefit (11 months). Finally, we considered the criteria of declaration of any monetary value from the BFP to be a beneficiary, but it was not possible to confirm this information with the national database of beneficiaries, as the identification of the respondents of the HBS is confidential. However, there is no reason to believe that a person would declare to be beneficiary if he/she is not, but the contrary is possible, which would underestimate the results of our study. In this case, the impact of the BFP may be higher.

Experimental studies are considered the gold standard for impact evaluation, since they theoretically provide results without residual or unmeasured confounding [36]. The conduction of an experimental study in Brazil would not be viable for ethical reasons, since it would not be possible to randomize the families to receive the benefit or not, neither to control the entire causal path between income transfer and diet changes [37]. On the other hand, the quasi experimental method (previously adopted to assess the BFP impact on other outcomes [6, 37]), with national representative data, tends to obtain robust results on causal effects due to the use of large databases [38]. In Mexico, an experimental study conducted in the second phase of the CCTP implementation was compared with another one using propensity score matching. It was concluded that this method was able to remove the vast majority of errors, and obtain reasonable estimates of impact [39].

## Conclusions

This study brought robust results about the *Bolsa Família* Program positive impacts on food purchases and diet quality in Brazil, indicating higher food expenses and higher caloric availability among beneficiary households, mainly from *in natura* and minimally processed foods. In this sense, this study contributes to confirm the capacity of cash transfer programs to improve the autonomy and empower the most vulnerable populations so that they can choose the more appropriate way to spend and invest their money.

However, the small magnitude of the changes observed in the food profile consumed between the groups indicate the existence of access restrictions to greater variety of foods for low-income families. Only the income increase is no guarantee of effective improvement in the family’s diet. Other public policies to promote the access to *in natura* or minimally processed foods at affordable prices to low-income people are key to ensuring a proper and healthy diet for all.

The recent practice of evaluating public policies is growing in Brazil [40]. The formulation and implementation of public policies based on high quality scientific evidence can ensure that policy-making is as reasoned and correct as possible [41]. Therefore, it is a challenge for both researchers and politicians to obtain the best evidence possible, which should be translated into effective measures to improve the population’s health.

## Abbreviations

BFP: *Bolsa Família* Program
CCTP: Conditioned cash transfer programs
HBS: Household budget survey

## Footnotes

1 Ministry of Social Development. Available at: http://mds.gov.br/assuntos/bolsa-familia/o-que-e (Accessed on August 2015).

2 Ministry of Social Development. Synthesis information report of social programs [Relatório de Informações Síntese dos programas sociais]. Available at: http://aplicacoes.mds.gov.br/sagi/RIv3/geral/index.php (Accessed on August 2015).

3 IBGE- Instituto Brasileiro de Geografia e Estatística. Pesquisa de Orçamentos Familiares 2008–2009: Despesas, rendimentos e condições de vida. Rio de Janeiro: IBGE; 2010.

4 IBGE- Instituto Brasileiro de Geografia e Estatística. Pesquisa de Orçamentos Familiares 2008–2009: Avaliação nutricional da disponibilidade de alimentos no Brasil. Rio de Janeiro: IBGE; 2010.

5 IBGE- Instituto Brasileiro de Geografia e Estatística. Estudo Nacional das Despesas Familiares- ENDEF: 1974–1975. Rio de Janeiro: IBGE; 1978.

6 Núcleo de Estudos e Pesquisa em Alimentação da UNICAMP. Tabela Brasileira de Composição de Alimentos – TACO. versão 1. Campinas: NEPA/UNICAMP; 2004.

7 United States Department of Agriculture. USDA National Nutrient Database for Standard Reference, Release SR23. Washington, DC: USDA; 2010.

8 Khandker SR, Koolwal GB, Samad HA. Handbook on impact evaluation quantitative methods and practices. Washington, DC: The World Bank; 2010.

9 Baptistella JCF. Avaliação de programas sociais: uma análise do impacto do Bolsa Família sobre o consumo de alimentos e status nutricional das famílias. Dissertação de Mestrado, Programa de Pós-graduação em Economia Aplicada da Universidade Federal de São Carlos. São Carlos: UFSC; 2012.

10 Brasil, Ministério do Desenvolvimento Social e Combate à Fome; Secretaria de Avaliação e Gestão da Informação. Primeiros resultados da análise da linha de base da pesquisa de avaliação de impacto do Programa Bolsa Família. Brasília: MDS; Cedeplar; 2007.
